# Supplementary material for: Association between oral microbiome and seven types of cancers in East Asian population: a two-sample Mendelian randomization analysis
Source: Front Mol Biosci. 2023 Nov 21;10:1327893. doi: 10.3389/fmolb.2023.1327893 (PMC10702768; doi:10.3389/fmolb.2023.1327893)
Supplement: Supplementary file 1 [file DataSheet1.ZIP › Supplementary Materials/MR plots for tongue/tongue═╝/Gastric cancer/pheno.183_to_gastric cancer_leave_one_out.pdf]

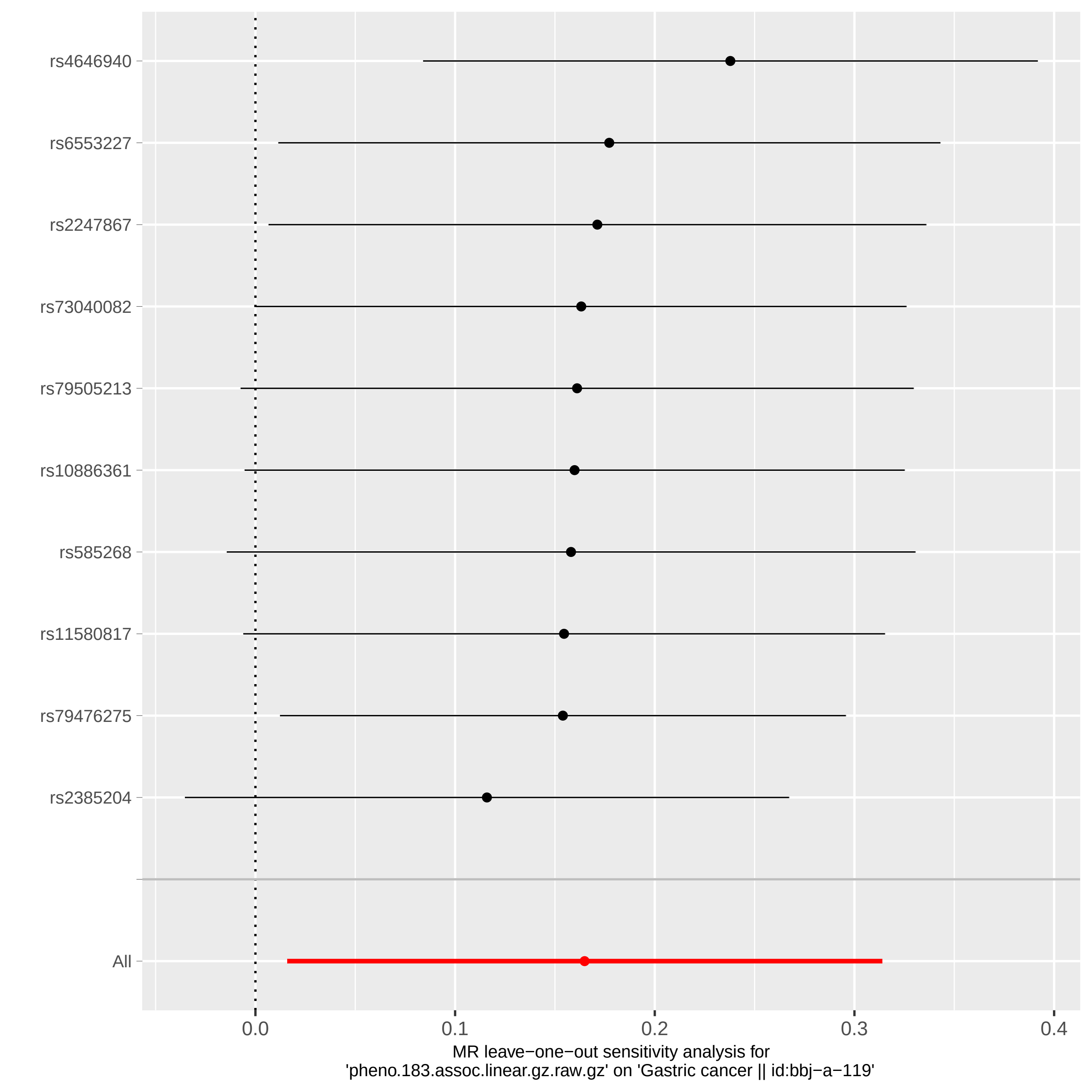

rs4646940
rs6553227
rs2247867
rs73040082
rs79505213
rs10886361
rs585268
rs11580817
rs79476275
rs2385204
All
0.0
0.1
0.2
0.3
0.4
MR leave−one−out sensitivity analysis for
'pheno.183.assoc.linear.gz.raw.gz' on 'Gastric cancer || id:bbj−a−119'
